# Moroccan women and tattoos: characteristics, perception and stigmatization

**DOI:** 10.1192/j.eurpsy.2025.2434

**Published:** 2025-08-26

**Authors:** A. Souidi, S. Stati, S. Belbachir, A. Ouanass

**Affiliations:** 1 Ar-razi hospital, Sale; 2 psychiatry B, Ar-razi hospital, Salé, Morocco

## Abstract

**Introduction:**

Tattooing is a mode of bodily expression that belongs to both tradition and modernity. This form of expression is rich in variant and variable meanings for women in general, and women with psychiatric disorders in particular. Their popularity and prevalence are increasing rapidly, particularly among young people. However, despite this expansion, the social perception of tattoos, particularly among women, remains a complex and often stigmatized subject. Hence the interest in knowing the characteristics of tattoos among women in Morocco, the different perceptions of the latter as well as the correlation of tattooing with psychiatric disorders.

**Objectives:**

To study the characteristics, meaning and perception of tattoos among Moroccan women, past and present. In an attempt to better understand the potential health implications of this mode of expression and its correlation with mental health.

**Methods:**

A descriptive cross-sectional study was carried out among women of different profiles. Data were collected using a form sent online and distributed in paper form. The form collected socio-demographic data, descriptive characteristics of the tattoos and the women’s mental health status. Statistical analysis was performed using Jamovi 2.3.

**Results:**

Our results focused on the socio-demographic characteristics of our population, it was carried out on 52 women, 57.7% of whom were aged between 25 and 40, 62% of our sample were single and the majority(92%) had a level of education higher than the baccalaureate. Also, almost 30% were unemployed. ours study results focused essentially on the tattoo characteristics, summarized in Image 1. Image 2 shows the results concerning the psychiatric history of our population.

**Image 1:**

**Figure:**
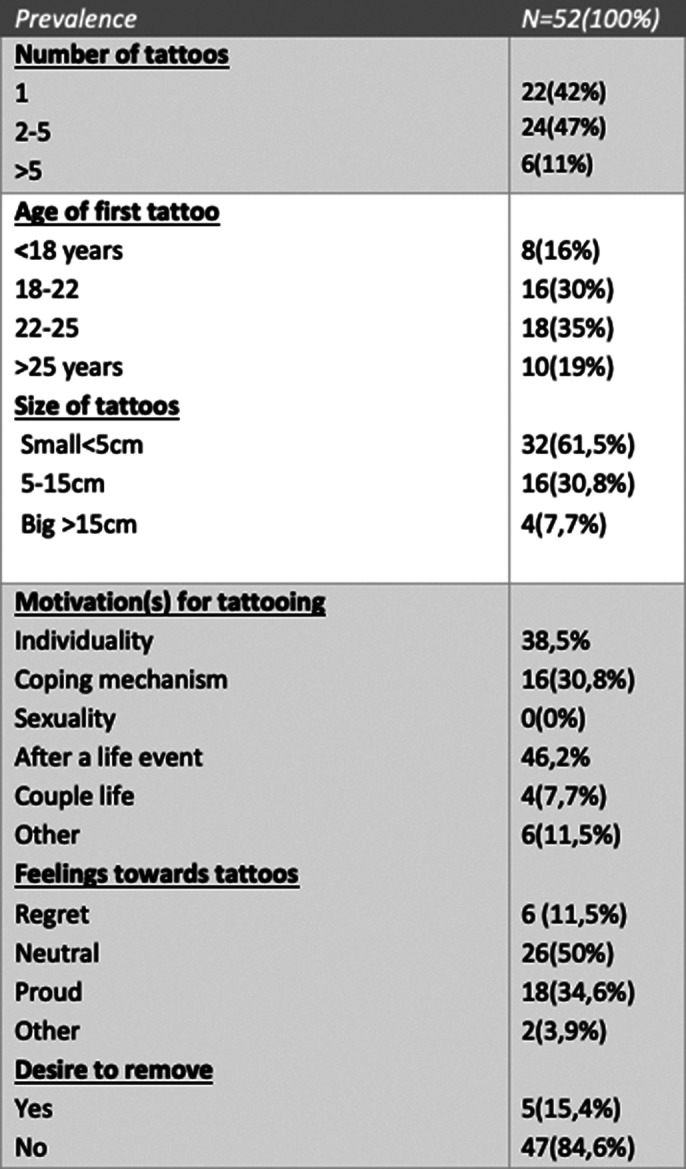

**Image 2:**

**Figure:**
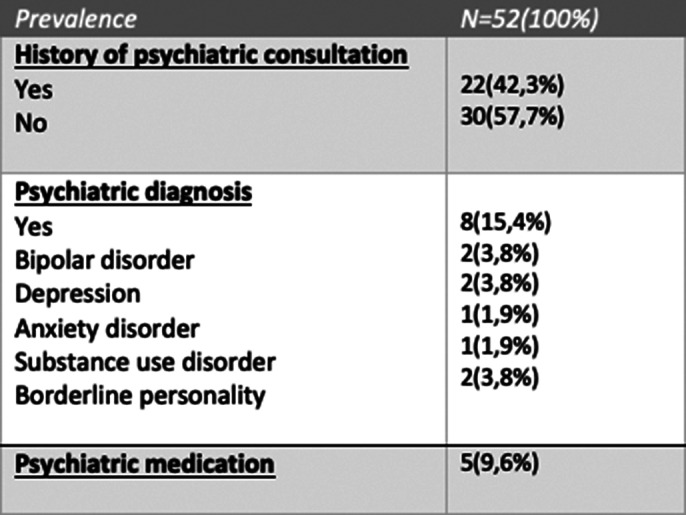

**Conclusions:**

Tattooing is a means of expression and a form of body modification, rich in cultural and personal significance, with a dual affiliation to tradition and modernity. It is highly prevalent among women, and has implications for both mental and physical health.

**Disclosure of Interest:**

None Declared

